# CALHM2 is a mitochondrial protein import channel that regulates fatty acid metabolism

**DOI:** 10.21203/rs.3.rs-4985689/v1

**Published:** 2024-09-13

**Authors:** Elizabeth Jonas, Nelli Mnatsakanyan, Felix Rivera-Molina, Andrew Robson, Alexandra MacColl Garfinkel, Amrendra Kumar, Stephen Batter, Valeria Padovano, Kaitlin Webster, Rebecca Cardone, Justin Berg, Derek Toomre, Richard Kibbey, Michael Caplan, Mustafa Khokha

**Affiliations:** Yale Medical School; Penn State College of Medicine; Yale University School of Medicine; Yale University School of Medicine; Yale University; Penn State College of Medicine; Yale University School of Medicine; Yale University; Yale School of Medicine; Yale University School of Medicine; Yale School of Medicine; Yale University; Yale University School of Medicine; Yale University; Yale University

## Abstract

For mitochondrial metabolism to occur in the matrix, multiple proteins must be imported across the two (inner and outer) mitochondrial membranes. Classically, two protein import channels, TIM/TOM, are known to perform this function, but whether other protein import channels exist is not known. Here, using super-resolution microscopy, proteomics, and electrophysiological techniques, we identify CALHM2 as the import channel for the ECHA subunit of the mitochondrial trifunctional protein (mTFP), which catalyzes β-oxidation of fatty acids in the mitochondrial matrix. We find that CALHM2 sits specifically at the inner mitochondrial and cristae membranes and is critical for membrane morphology. Depletion of CALHM2 leads to a mislocalization of ECHA outside of the mitochondria leading to severe cellular metabolic defects. These defects include cytosolic accumulation of fatty acids, depletion of tricarboxylic acid cycle enzymes and intermediates, and reduced cellular respiration. Our data identify CALHM2 as an essential protein import channel that is critical for fatty acid- and glucose-dependent aerobic metabolism.

## Introduction

Mitochondria have numerous critical functions in cellular energetics. They are the powerhouses of eukaryotic cells, using energy from the oxidation of nutrients such as fatty acids to regenerate ATP. Mitochondrial function is closely linked to its complex structure. Each mitochondrion has two (outer and inner) membranes, which together partition the organelle into an intermembrane space and a central matrix. The matrix side of the inner membrane is the site of fatty acid oxidation (β-oxidation), the primary metabolic pathway for the conversion of fats into energy^1 2^. β-oxidation liberates acetyl-CoA that can then enter the tricarboxylic acid (TCA) cycle. Reduced products made in the TCA cycle are oxidized in the electron transport chain, which results in the translocation of protons across the inner mitochondrial membrane into the intermembrane space creating an electrochemical gradient. The ATP synthase uses this gradient to generate ATP. Therefore, the inner mitochondrial membrane is critical for energy production^3,4^. Multiple β-oxidation enzymes are associated with the inner membrane, including the mitochondrial trifunctional protein (mTFP), which catalyzes three of the four mitochondrial steps of fatty acid oxidation^5–8^.

The mTFP and many other inner membrane and matrix proteins are nuclear encoded and translated in the cytosol; therefore, they must be imported across the two mitochondrial membranes. A canonical import pathway has been well-defined where protein precursors with mitochondrial targeting presequences are imported by two ion channels: TOM (the translocase of the outer membrane) and TIM23 (the inner membrane translocase subunit)^9–14^. Once in the inner membrane or matrix, a mitochondrial processing peptidase removes the presequences, and chaperones take over to refold the proteins into their three-dimensional structures^15–17^. Many of the foundational studies defining TIM/TOM targets were performed in yeast, where β-oxidation of fatty acids occurs in the peroxisome not the mitochondria^18^. This opens the possibility that metazoans may require an evolutionarily divergent system to transport β-oxidation enzymes into the mitochondria.

Here, we show that CALHM2 localizes to the inner mitochondrial membrane and acts as an import channel for the ECHA subunit of the mTFP. CALHM2 resembles a connexin channel that is well conserved across vertebrates but has not been assigned a function^19,20^. We now show that CALHM2 can independently translocate ECHA across a lipid bilayer. Loss of CALHM2 results in the mislocalization of ECHA to the cytosol, which leads to an accumulation of cytosolic fatty acids. The loss of CALHM2 and mislocalization of ECHA result in severe metabolic compromise, impairing ATP production and reducing mitotic rates. These findings highlight the indispensable role of CALHM2 in importing ECHA to the matrix and define a pathway, distinct from TIM/TOM, for protein import into the mitochondria.

## Results

### CALHM2 is localized to the inner mitochondrial membrane

**Figure 1:**
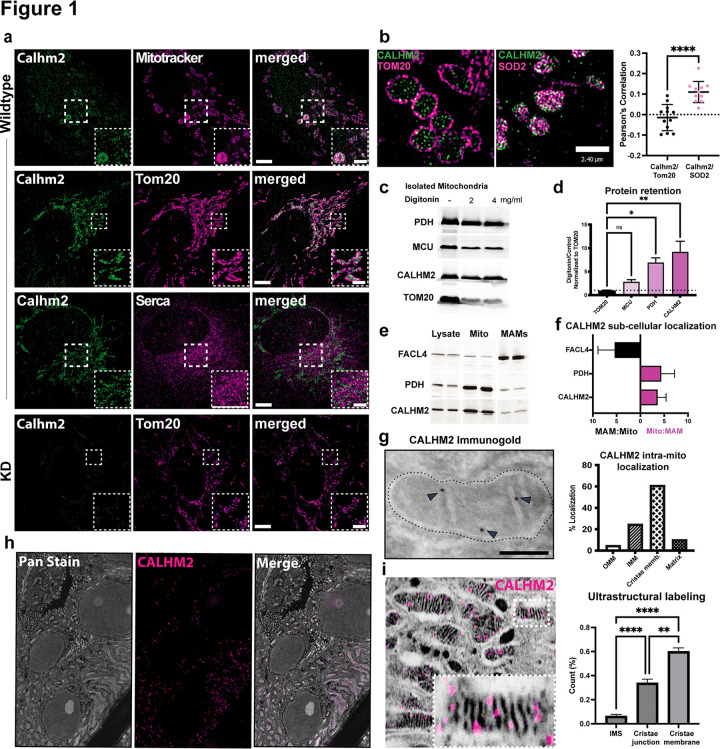
CALHM2 localizes to the inner mitochondrial membranes a) Immunofluorescence staining to co-localize CALHM2 with intracellular compartments in RPE cells. CALHM2, green; mitochondrial (MitoTracker and TOM20) and ER (SERCA), magenta. In CALHM2 KD2 RPE cells (bottom row), CALHM2 signal is reduced demonstrating specificity of the anti-CALHM2 antibody. Scalebars 5 μm (2 μm in insets). b) Representative structural illumination (SIM) images showing immunofluorescence staining for CALHM2 and the outer mitochondrial membrane marker TOM20 (left) and the inner mitochondrial matrix protein SOD2 (right) in RPE cells. Right panel graph: Pearson’s correlation for CALHM2 and TOM20/SOD2. **** p<0.0001; t test. Scale bars =2.40 μm. c) Western blot analysis of total mitochondrial lysate after exposure to the indicated concentrations of digitonin to remove the solubilized outer mitochondrial membrane. TOM20 localizes to the outer mitochondrial membrane, MCU localizes to the inner mitochondrial membrane, PDH localizes to the matrix. CALHM2 is retained as well as MCU or PDH upon outer membrane removal, indicating its localization to the inner membrane or matrix compartment. d) Remaining protein levels after digitonin treatment and outer membrane removal. Protein amounts were normalized to TOM20. Mean ± S.E.M., n=3. *p <.05; **p<.01; one-way ANOVA. e) Western blot analysis of total lysate, mitochondria, and MAMs fractions. CALHM2 is enriched in mitochondria but not in MAMs. The identity of the fractions was confirmed with antibodies to PDH (mitochondria) and FACL4 (MAMs). f) Quantification of CALHM2 membrane localization in mitochondria vs MAMs. g) CALHM2 immunogold labeling indicates localization to mitochondrial inner membrane cristae (scale bar 0.2 μm). h) Widefield PanExM images of renal tubule epithelial cells indicate mitochondrial localization of CALHM2. i) Higher magnification PanExM shows localization of CALHM2 primarily to the inner membrane cristae, cristae junctions (CJ), and the inner boundary membrane (IMS). Bar graph of CALHM2 signal at specific locations in the mitochondria (right). **p<.01; ****p<0.0001; one-way ANOVA.

To ascertain the function of CALHM2, we first sought to determine its localization in the cell. We assessed the endogenous subcellular localization of human CALHM2 in human telomerase reverse transcriptase (hTERT) immortalized human retinal pigment epithelial (RPE) cells by structured-illumination microscopy. We found that CALHM2 co-localized with the mitochondrial-specific dye MitoTracker and TOM20 (Fig 1a). CALHM2 does not appear to co-localize with the ER marker anti-Sarcoendoplasmic Reticulum Calcium ATPase (SERCA) (Fig 1a).

Further analysis of the structured-illumination imaging revealed that CALHM2 is enveloped by the TOM20 staining and overlaps with superoxide dismutase 2 (SOD2), a component of the mitochondrial matrix (Fig 1b). Quantitative co-localization analysis using Pearson’s Correlation showed a higher correlation between SOD2 and CALHM2 than CALHM2 and TOM20 (Fig 1b), supporting the view that CALHM2 is at the inner membrane. To confirm that our anti-CALHM2 antibody signal is specific, we generated three independent RPE cell lines for CALHM2 knock-down (KD), targeting two distinct sites of the CALHM2 gene (Lines 1.1 and 1.2 target the same site, while Line 2 targets a second site, Ext Data Fig 1). In these cell lines, the CALHM2 signal is reduced confirming the specificity of our antibody (Fig 1a, Ext Data Fig 1).

Next, we studied the submitochondrial localization of CALHM2. We purified mitochondria from RPE cells and treated them with different concentrations of digitonin to remove the outer mitochondrial membranes. In this assay, proteins from the outer mitochondrial membrane are lost differentially compared to proteins in the inner membrane or matrix. We used the following proteins as markers of different mitochondrial compartments: TOM20 and mitochondrial calcium uniporter (MCU) for the outer and inner mitochondrial membranes, respectively, and PDH for the matrix. Treatment with digitonin reduced TOM20 levels substantially, indicating removal of the outer membrane, while reduction in MCU levels was less, and PDH was relatively preserved (Fig 1c, d). CALHM2 was well preserved compared to TOM20, suggesting that it is not localized in the outer membrane. CALHM2 levels were more comparable to MCU and PDH. Next, we assessed subcellular localization to mitochondrial-associated membranes (MAMs) via biochemical fractionation (Fig 1e,f). CALHM2 and the mitochondrial matrix protein PDH are enriched in the mitochondrial fraction, while Long-chain-fatty-acid-CoA ligase 4 (FACL4) is enriched in the MAM fraction (Fig 1e,f). Together, these data allow us to conclude that CALHM2 is localized to the inner mitochondrial membrane.

For greater structural resolution, we performed electron and expansion microscopy to reveal if CALHM2 is localized to the inner boundary membrane, the cristae membranes, or the cristae junctions. Immunogold labeling revealed that CALHM2 is most frequently distributed in cristae membranes (Fig 1g). Using expansion microscopy, we confirmed that CALHM2 is not localized to the plasma membrane and is exclusively in the mitochondria at low magnification (Fig 1h). At high magnification, CALHM2 is found most frequently at cristae and cristae junctions (Fig 1i).

### CALHM2 binds to the mTFP and regulates mTFP levels

**Figure 2:**
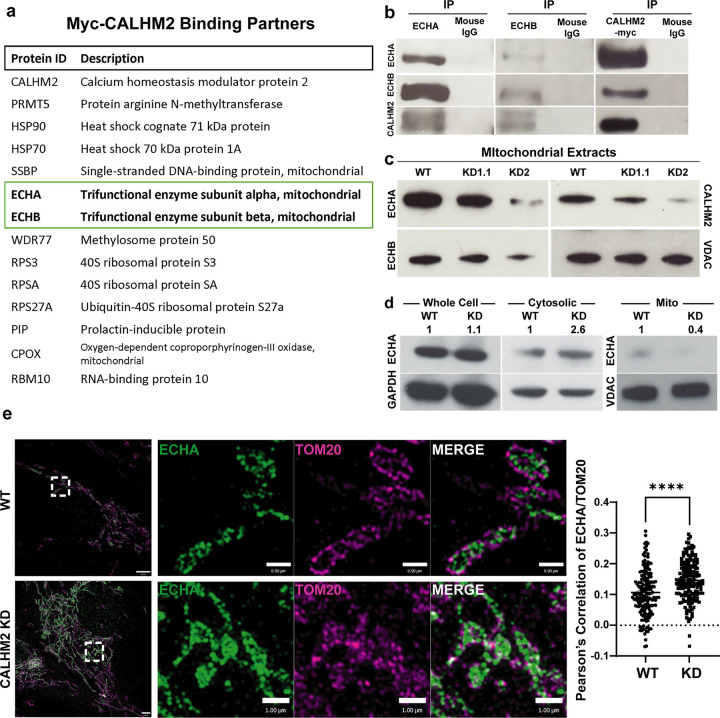
CALHM2 interacts with the mTFP and is required for ECHA localization a) Mitochondrial immunoprecipitation with anti-Myc antibody followed by LC-MS/MS analysis. Interacting proteins listed, indicating that mTFP α (ECHA) and β (ECHB) subunits interact with CALHM2. b) ECHA and ECHB co-immunoprecipitate with CALHM2 and each other. CALHM2-myc was transfected into HEK293 cells and co-immunoprecipitated with ECHA and ECHB. Immunoblots are labeled with the indicated antibodies. c) Immunoblots of mitochondrial extracts using the indicated antibodies reveal reduced mTFP subunits in CALHM2 KD RPE cells. VDAC acts as loading control for mitochondria. d) Immunoblot analysis of ECHA in whole cell, cytosolic, and mitochondrial fractions from WT and CALHM2 KD RPE cells. GAPDH is loading control for cytosolic fraction and VDAC is for mitochondrial fraction. Quantification of protein levels (numbers above blot) are normalized for GAPDH (cytosolic and whole cell fractions) and VDAC (mitochondrial fractions). e) Top row: representative structural illumination (SIM) images showing ECHA (green) localization within the mitochondria surrounded by the outer membrane stained for TOM20 (magenta) in WT cells. Bottom row: in CALHM2 KD cells, there is increased accumulation of ECHA with TOM20 outside of, or co-localizing with, the mitochondrial outer membrane (insets). Pearson’s correlation analysis shows a significant increase in TOM20 and ECHA colocalization in CALHM2KD relative to WT cells (****p<0.0001; Two tailed t-test).

To begin elucidating a role for CALHM2 in mitochondria, we expressed a CALHM2-Myc construct in RPE cells, immunoprecipitated the myc epitope, then performed liquid chromatography mass spectrometry (LC-MS/MS) to identify associated proteins. Surprisingly, amongst the top binding partners of CALHM2 were ECHA and ECHB, subunits of the mTFP (Fig. 2a, Ext Data Table 1). The mTFP is a hetero-octamer, with two genes, *HADHA* and *HADHB*, that encode the a (ECHA) and β (ECHB) subunits of the mTFP, respectively. Together these two subunits of the mTFP perform three consecutive steps in β-oxidation: 2-enoyl-CoA hydratase activity, an NAD^+^-dependent 3-hydroxyacyl-CoA dehydrogenase activity, and a CoA-dependent 3-ketothiolase activity. These steps culminate in the production of acetyl-CoA that is fed into the TCA cycle to produce NADH and FADH_2_ for oxidation in the electron transport chain.

To verify that CALHM2 binds to the mTFP, we performed reciprocal immunoprecipitation and western blotting (Fig 2b). With IP of either CALHM2, ECHA, or ECHB, we could detect each of the other proteins that are not present in an IgG control. Interestingly, while both subunits of the mTFP and two chaperone proteins (HSP90 and HSP70) were detected in this LC-MS/MS analysis, other proteins of the inner mitochondrial and cristae membranes such as components of the electron transport chain were not detected (Extended data Table 1). Struck by the mitochondrial localization of CALHM2, the association of CALHM2 with both subunits of the mTFP (ECHA, ECHB), and the importance of the mTFP in fatty acid metabolism, we focused our analysis on the relationship between ECHA, ECHB, and CALHM2.

Next, we wondered if CALHM2 regulates mTFP protein levels or function. We isolated mitochondria and examined protein levels in two different CALHM2 KD lines (Fig 2c). Testing the mitochondrial extracts, we noted reduced levels of ECHA and ECHB in both KD cell lines (Fig 2c). To evaluate whether ECHA was mislocalized as opposed to simply reduced in level, we compared the cytosolic fraction to our mitochondrial fraction. Interestingly, ECHA appeared relatively elevated in the cytosolic fraction compared to the mitochondrial fraction in CALHM2 KD cells vs. WT (Figure 2d). This result suggests that CALHM2 may be required for the proper localization of the mTFP to the mitochondria.

To better address the localization of ECHA in response to CALHM2 KD, we returned to our structured illumination immunofluorescence studies. We compared the localization of ECHA to TOM20, present in the mitochondrial outer membrane. In WT cells, ECHA appears to be surrounded by the TOM20 mitochondrial signal (Figure 2e – see merged channel). However, in the CALHM2 KD cell line, ECHA appears to be mislocalized and the geometry relative to TOM20 is not preserved. In this case, quantitative co-localization analysis using Pearson’s Correlation showed a higher correlation between TOM20 and ECHA in CALHM2 KD cells compared to WT indicating that ECHA was mislocalized (Fig 2e).

### CALHM2 affects mitochondrial cristae and fatty acid levels

**Figure 3:**
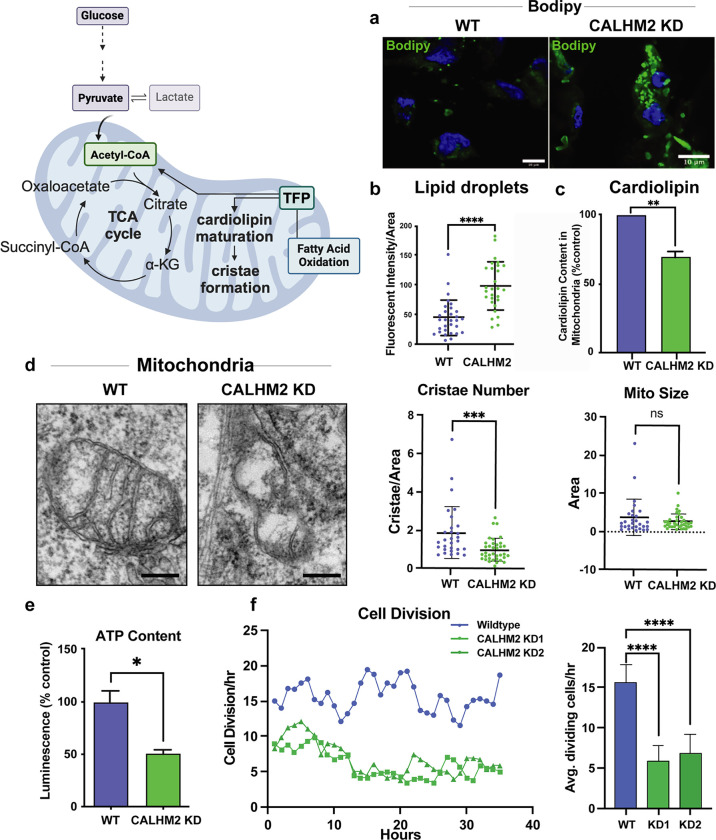
Mitochondrial morphology is abnormal in CALHM2 KD cells a) Representative images of the BODIPY stained lipid droplets (green) in WT and CALHM2 KD cells. b) Total quantification of BODIPY intensity in WT and CALHM2 KD cells **** p<0.0001; ANOVA with Fisher’s *post hoc* test. c) Total cardiolipin content is decreased in CALHM2KD RPE cell mitochondria compared to WT. ** p≤.01; unpaired t-test; Scale bars = 10 μm. N = 2. d) Representative electron microscopic images of mitochondria from WT and CALHM2 KD RPE cells. CALHM2KD cells have mitochondria with reduced numbers of cristae compared to WT cells although the mitochondrial size is not changed. n=30 mitochondria, *** p=0.001; Student’s t test. Scale bars = 0.2 μm. e) ATP levels are decreased in CALHM2KD cells compared to WT as measured by firefly Luciferin/luciferase luminescence. Equal amounts of mitochondrial protein were used for each cell type. Luminescence level was normalized to wild-type level, * p<0.05; Two Samples t-test. N = 3 replicates. f) Cell division rate is decreased in CALHM2KD cells compared to that of WT. Average number of dividing cells per hour was calculated from a 36 hr phase contrast imaging time lapse. Error-bars represent standard deviation, **** p<0.0001; Two Sample t-test.

If CALHM2 is necessary for mTFP mitochondrial localization and function, we expected CALHM2 KD cells to exhibit abnormal fatty acid metabolism as previously described in mTFP disease and *HADHA* mutant and KD cells^21^. BODIPY is a commonly used indicator of intracellular neutral lipid levels. We found that CALHM2 depleted cells have an increase in the BODIPY signal (Fig 3a,b), suggesting an accumulation of these lipids in the cytosol.

In addition to its role in the mTFP, the α subunit of the mTFP (ECHA) acts as an acyltransferase in cardiolipin remodeling^21^. Cardiolipin is an essential diphosphatidylglycerol lipid in the inner mitochondrial membrane that plays a critical role in creating normal cristae structures and positioning of inner membrane transport complexes^22,23^. Cardiolipin was reduced in CALHM2-depleted mitochondria compared to WT (WT) (Fig. 3c), further evidence that mTFP function was impaired.

Cardiolipin is specific to the inner membrane making up 20% of the lipid content. Therefore, reduction of cardiolipin is likely to affect mitochondrial inner membrane structure. We examined mitochondrial ultrastructure by transmission electron microscopy of CALHM2 KD cells and identified abnormalities in cristae morphology (Fig 3d). In WT cells, the mitochondria are oblong with characteristic cristae formed by the inner membrane. In the majority of CALHM2 KD cells, the number and size of the mitochondria are normal (Fig. 3d and Ext Data Fig 2); however, the mitochondria have a reduced number of cristae as predicted by the decrease in cardiolipin levels (Fig 3d). Altogether, we conclude that CALHM2 is essential for inner mitochondrial membrane structure and cardiolipin levels in addition to normal lipid metabolism.

Due to the disruption of mitochondrial cristae and a presumed failure to metabolize fatty acids, we predicted that cellular ATP content should be low in CALHM2 depleted cells. We found that ATP levels of CALHM2 KD cells are approximately 50% of the WT levels (Fig 3e). In the context of abnormal lipid metabolism and a drop in ATP levels, we wanted to assess the overall health of these cells via cell division rate. We counted the number of cells in mitosis per hour. Over a 36 hr window, the number of cell divisions in CALHM2 KD cells was roughly a third of the number in WT cells (Fig 3f), suggesting a global growth defect.

### CALHM2 affects cellular respiration

**Figure 4:**
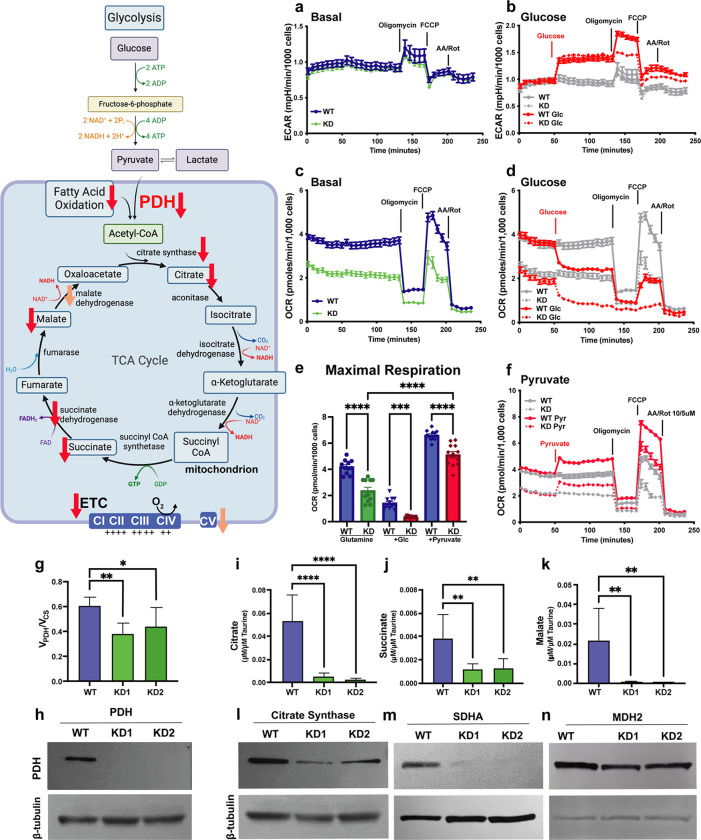
CALHM2 is essential for normal mitochondrial metabolism a) Graph showing changes in extracellular acidification rate over time (ECAR) as measured by Agilent Technologies XF96 Pro Analyzer. WT or CALHM2 KD cells were cultured in XF Assay Medium containing 2 mM Glutamine and 0 glucose. ECAR was evaluated during addition of the following reagents at times indicated on the graph: 5 μM oligomycin, 20 μM FCCP, 5 μM rotenone plus 10 μm antimycin A (n = 11). b) ECAR before and after addition of 5 mM glucose at the indicated time point (red traces). The gray graphs from panel A are shown again for comparison (n = 11). c) Oxygen consumption rate (OCR) as measured by Agilent Technologies XF96 Pro Analyzer in RPE cells supplemented with 2 mM glutamine 0 glucose. Oligomycin, FCCP and rotenone plus antimycin A were added at times indicated (n = 11). d) OCR before and after the addition of 5 mM glucose at the indicated time. e) Quantification of maximal respiration (FCCP) with 2 mM glutamine in all conditions as well as additions as indicated (n = 11); *** p<0.001; **** p<0.0001. f) OCR before and after the addition of 5 mM pyruvate at the indicated time (red lines). The gray graphs from panel C are shown again for comparison g) Relative contributions of maximal enzymatic rate of pyruvate dehydrogenase PDH (V_PDH_) to maximal enzymatic rate of citrate synthase (V_CS_) in WT and CALHM2 KD cells. *p<0.05, **p≤0.01; one-way ANOVA. Enzymatic rates were measured by labeled C13 uptake over time (n = 6). h) Representative (n = 3 per cell line) immunoblot showing reduced PDH protein levels in CALHM2 KD cells vs. WT cells. (β tubulin serves as control for protein loading). i) Total citrate relative to Taurine concentration in control and CALHM2 KD cells; ****p<0.0001. j) Total succinate relative to Taurine concentration in control and CALHM2 KD cells. **p≤0.01. k) Total malate concentration relative to Taurine in control and CALHM2 KD cells; **p≤0.01. l) Representative immunoblot showing reduced level of citrate synthase protein from whole cell lysates. (β tubulin serves as control for protein loading). m) Representative immunoblot showing reduced level of SDHA protein from whole cell lysates. (β tubulin serves as control for protein loading). n) Representative immunoblot showing reduced level of MDH2 protein from whole cell lysates. (β tubulin serves as control for protein loading).

The TCA cycle generates energy via the oxidation of acetyl-CoA that can be derived from carbohydrates during glycolysis, fatty acids during β-oxidation, or proteins during amino acid catabolism. We predicted that a loss of CALHM2 would lead to a reduction in fatty acid metabolism as mTFP no longer localizes to the mitochondrial matrix. When β-oxidation is inhibited, glycolysis may compensate by producing acetyl-CoA from pyruvate (Fig 4, illustration). This process results in the acidification of the extracellular medium due to the production of lactate, which can be measured as the Extracellular Acidification Rate (ECAR).

To determine how CALHM2 KD cells use glycolysis compared to WT cells, we measured ECAR in the presence and absence of glucose. In the absence of glucose, we observed no difference in the ECAR of WT and CALHM2 KD cells, indicating similar overall rates of glycolysis under these conditions (Fig 4a). Upon the addition of glucose (5 mM, Fig 4b), we observe an equivalent increase in ECAR between the two groups, suggesting glycolysis is not increased in response to the reduction of β-oxidation in CALHM2 KD cells (overlapping red lines in Fig 4b after glucose addition compared to gray lines which are reproduced from Fig 4a). Strikingly, the addition of the ATP synthase inhibitor oligomycin to WT cells approximately doubles their ECAR response to glucose, revealing a higher glycolytic capacity. However, in CALHM2 KD cells, exposure to oligomycin does not elevate the acidification rate any further (Fig 4b, split in red lines after oligomycin), showing a diminished response of glycolytic acidification to ATP synthase inhibition. These data suggest that CALHM2 KD cells are either at their maximal glycolytic capacity and cannot respond to the oligomycin-induced loss of ATP production or that CALHM2 KD cells have a reduced demand for ATP synthesis under these conditions.

To understand the mitochondrial respiratory rate and capacity of CALHM2 KD cells, we next assessed oxygen consumption rate (OCR) in response to various metabolic substrates. In the absence of glucose (2 mM glutamine), baseline and protonophore (FCCP)-stimulated respiratory capacity are decreased by nearly half in CALHM2-depleted cells compared to WT (Fig 4c, e), consistent with a reduction in oxidative capacity. In 5mM glucose-containing media, both cell lines strongly suppress their respiration. Respiration is almost completely inhibited by glucose in CALHM2 KD cells compared to a less severe reduction in WT cells (Fig 4d, e, compare red lines to gray). This reduction in OCR shows that both WT and CALHM2 KD shift toward glycolytic ATP production in the presence of glucose; however, the relative difference between them indicates that respiratory capacity is markedly lower in CALHM2 KD cells.

Since CALHM2 KD cells seem to be at their maximal glycolytic capacity in 5mM glucose, we sought to bypass glycolysis by providing either pyruvate or lactate (in No glucose) to determine if respiration could be rescued (Fig 4 illustration). The acute response and maximal capacity of WT cells to lactate are enhanced over CALHM2 KD (Ext Data Fig 3a-c). This suggests that either LDH or redox shuttling is limiting the use of lactate for acetyl-CoA production in CALHM2 KD cells. Interestingly, upon the addition of pyruvate, CALHM2 KD cells increase respiration comparably to WT cells but do not reach WT levels (Fig 4 e,f, comparing dotted red to solid red lines after pyruvate). Additionally, the maximal respiratory rate (FCCP response) is still lower in CALHM2 KD cells, consistent with a diminished respiratory capacity (Fig 4e). In summary, while CALHM2 cells can respond to pyruvate, they still have a diminished respiratory capacity suggesting that downstream metabolism may be affected such as the TCA cycle or electron transport.

PDH is localized to the mitochondrial matrix and links glycolysis to the TCA cycle by converting pyruvate into acetyl-CoA (Fig 4, illustration). As our data indicate that CALHM2 KD cells have a disrupted oxidative response to endogenous glycolytic products, we sought to characterize the levels and activity of PDH and essential TCA enzymes. To address step-by-step defects in glycolytic and mitochondrial metabolism, including PDH activity, we performed Mass Isotopomer MultiOrdinate Spectral Analysis (MIMOSA) on WT and CALHM2 KD cells. This technique uses the incorporation of mass [U-^13^C_6_]-D-glucose in place of the unlabeled forms for analysis of labeled products by LC-MS/MS^24^. The relative rates of production of matrix acetyl-CoA from pyruvate versus other sources such as β-oxidation are determined from the fractional contribution of pyruvate oxidation by the mitochondria (V_PDH_/V_cs_). PDH activity is significantly reduced in CALHM2 KD cells (Fig 4g) compared to WT. Indeed, PDH protein levels are significantly reduced in CALHM2 KD cells (Fig 4h).

We next measured TCA cycle intermediates by LC-MS/MS and found that CALHM2 KD cells have a dramatic decrease in concentration of all the measured metabolites (citrate, succinate, malate) (Fig 4i–k). These data suggest that there may be insufficient anaplerosis to maintain the TCA cycle metabolite pool. The related enzymes of the TCA cycle, citrate synthase, succinate dehydrogenase A (SDHA), and malate dehydrogenase (MDH2) are also reduced (Fig 4I–n).

Finally, we examined components of the electron transport chain to address the last steps of oxidative metabolism in the mitochondria. Complexes I, II, III, and V levels are decreased in CALHM2 depleted cells, although more markedly for CI and CII (Ext Data Fig 3d,e), supporting the conclusion that TCA cycle and ETC enzymes are downregulated.

In summary, CALHM2 KD cells are unable to efficiently utilize endogenous lactate and pyruvate to drive the TCA cycle and the ETC. Interestingly, we expected to see a defect primarily in β-oxidation of fatty acids but not glycolysis/TCA/ETC; however, our data highlight that CALHM2 is essential for these other metabolic processes.

### CALHM2 is a protein import channel for ECHA

**Figure 5:**
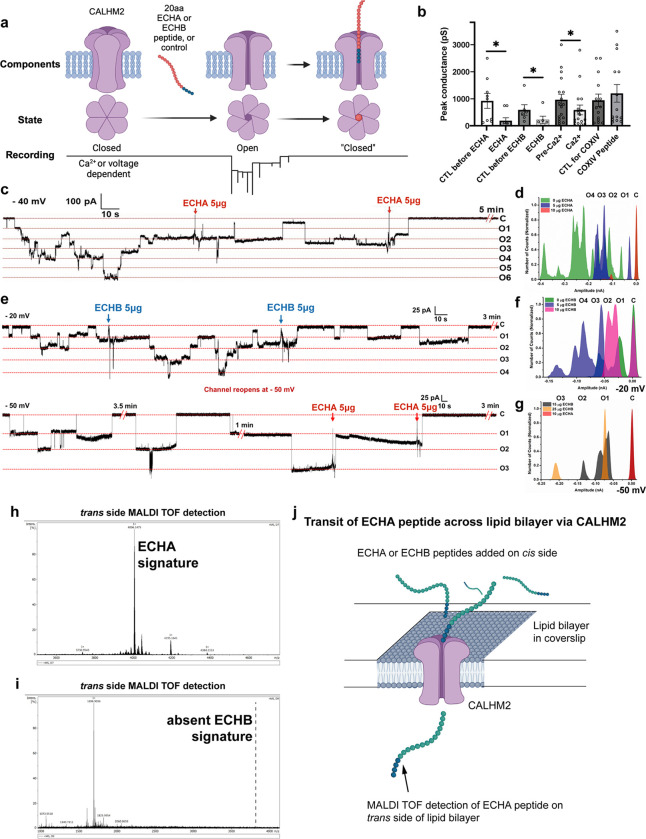
CALHM2 acts as a transporter for ECHA a) Illustration showing open and closed states of the CALHM2 channel interacting with 20 amino acid (aa) peptides. The left panel indicates the closed state, the middle the open state in the absence of peptide, the right panel indicates the inhibited state of the channel when peptide is inhibiting pore ion conductance. b) Quantification of peak channel conductance during excised patch proteoliposome recordings of reconstituted CALHM2 protein before and after the addition of the indicated reagents to the recording chamber. COXIV peptide serves as a control, non-interacting, peptide. ECHA: * p = .0125; ECHB: * p = .0111; Ca^2+^: * p = .0244. Two samples t-tests performed for each experiment presented. ECHA N = 9; ECHB N = 6; Ca^2+^ N = 17; COXIV N = 15 c) Representative recording of lipid bilayer reconstituted with CALHM2 protein. ECHA N terminal signaling peptide was added to the *cis* side of the bilayer at the indicated times and concentrations (/ml). Holding voltage and open and closed states are indicated. d) Amplitude histogram indicates dose-dependent modulation of CALHM2 channel activity upon the addition of increasing concentrations of ECHA peptide. e) Representative recording of lipid bilayer reconstituted with CALHM2 protein. ECHB N-terminal signaling peptide was added to the *cis* side of the bilayer at the indicated times and concentrations (/mL). Holding voltage and open and closed states are indicated. After recording at the initial holding voltage (−20 mV), the holding voltage was hyperpolarized (−50 mV) leading to the re-opening of the channel. ECHA peptide was then added, which closed the channel. f) Amplitude histogram indicates increasing CALHM2 channel closed states after the addition of increasing concentrations of ECHB peptide at the holding voltage of −20 mV for the top recording line. g) Amplitude histogram indicates CALHM2 channel closed state (in the presence of ECHB peptide) then open states after voltage change followed by increasing closed states after ECHA peptide addition. h) MALDI TOF – ECHA. The peak corresponding to the molecular weight of the peptide (~4006) was detected by MALDI TOF suggesting that the peptide was transported through the CALHM2 channel during the recording. i) MALDI TOF – ECHB. The molecular weight of the peptide is ~3828. No peak was detected at this position. j) Illustration showing the addition of peptide to *cis* side of the chamber during reconstituted lipid bilayer recording of CALHM2 protein. MALDI TOF analysis was performed on the solution removed from *trans* side of the bilayer chamber. The presence of ECHA but not ECHB peptide was detected by MALDI TOF on side B.

Our results thus far suggest that CALHM2 is localized to the mitochondria, regulates mTFP localization, and is essential for normal cristae structure, cardiolipin and fatty acid levels, and normal cellular respiration. Based on cryo-EM, CALHM2 is a connexin-like transmembrane channel^19,20^, and given the mislocalization of ECHA in our IF studies, we speculated that CALHM2 imports the mTFP to the matrix side of the inner membrane (Fig 5a).

To investigate the ion channel’s biophysical properties, we carried out proteoliposome and planar lipid bilayer recordings of purified reconstituted CALHM2. Previous whole-cell electrophysiology of overexpressed human CALHM2 showed that CALHM2 produces a robust voltage-dependent current in the absence of Ca^2+^ and is Ca^2+^ inhibited^19^. In keeping with this previous report, our single channel recordings demonstrate that CALHM2 forms a large conductance voltage-gated channel with multiple sub-conductance states and a peak conductance value of ~1 nanoSiemens (nS) (Fig. 5b, far left CTL before ECHA and Ext Data Fig. 4). Similarly to the whole cell currents reported^19^, CALHM2 forms a negatively rectifying channel which is inhibited by the addition of calcium to the bath during the recordings (Fig 5b, Ext Data Fig 4c).

Next, to test whether CALHM2 might be an import channel, we designed ECHA and ECHB N-terminal peptides and added them to the recording chamber during electrophysiological measurements. When the N-terminal ends of transiting mitochondrial proteins pass through import channels, they inhibit channel activity in a concentration dependent manner^13,25,26^ (Fig. 5a). Consistent with this notion, in patch-clamp recordings of proteoliposomes, we observed an inhibition of CALHM2 channel activity upon the addition of both the ECHA and ECHB peptides whereas a control peptide (N terminus of COXIV) had no effect (Fig. 5b and Ext Data Fig. 4a-d).

We examined the concentration dependence of the peptides on CALHM2 conductance in planar lipid bilayers. In a representative trace (Fig 5c), the first addition of the ECHA peptide significantly reduces the peak conductance of the channel and the number of subconductance states (Fig 5c - C is closed, O1-O3 are smaller, less open, subconductance states) and see amplitude histogram 5d (green to blue peaks). The second addition of the peptide completely inhibits channel conductance (Fig 5c,d, right end of trace, loss of O1-O6, amplitude histogram - blue to red peaks). The group data confirm that ECHA reduces CALHM2 conductance in a concentration dependent manner (Ext Data Fig 4e,f). In bilayer recordings, we failed to observe CALHM2 channel activity at positive voltages, consistent with the published report on whole cell currents (Ext Data Fig 4f,h)^19^.

In contrast to ECHA, in a representative recording with ECHB, the first addition of peptide fails to inhibit conductance, but instead increases the frequencies of transitions between subconductance states suggesting an interaction of ECHB with the channel but a failure to completely inhibit conductance (Fig 5e top panel and f green to purple). Further addition of ECHB reduces the probability of channel opening and peak conductance (Fig 5e top panel and f, Ext Data Fig 4g,h). To study if the interaction between CALHM2 and ECHA and ECHB peptides is voltage-dependent, we changed the voltage from −20 mV to −50 mV (compare top and bottom in Fig 5e and amplitude histograms f, g). The voltage change reopens the channel, although at a subconductance state. Subsequent additions of ECHA peptide completely inhibit channel conductance in a dose-dependent manner (Fig 5e bottom panel and g). These results indicate that ECHA is more efficient than ECHB at inhibiting CALHM2 conductance at the concentrations tested in our assay.

While electrophysiological recordings show an inhibition of CALHM2 channel activity with either peptide in a concentration-dependent manner, we could not distinguish between channel transit or inhibition (Fig 5j). Therefore, we used a low concentration (2.5 μg) of ECHA, where there was no discernible block of CALHM2. At higher concentrations (5 μg), the channel was partially inhibited (Ext Data Fig 4i). To test for transit of the peptide through the channel, we added the low concentration (2.5 μg) of ECHA to only the *cis* side of the lipid bilayer chamber, recorded channel activity, and then removed the solution from the *trans* side for MALDI TOF analysis (Fig 5h). The MALDI TOF trace confirmed the transit of the ECHA peptide from the *cis* to the trans side (Fig 5h,j). We did not detect the ECHB peptide on the *trans* side when we repeated this experiment with ECHB on the *cis* side (Fig 5i, Ext Data Fig 4j). This result was surprising, as we have shown ECHB directly interacts with CALHM2 in the IP assay and can inhibit channel activity (albeit less efficiently) in a concentration dependent manner.

Because of the differential transit of ECHA and B through the channel, we studied the charge distribution of the ECHA and ECHB presequence peptides. The alignment of amino acid sequences of ECHA and ECHB revealed that the former has more negatively charged amino acid residues and is more linear in structure, which could explain the differences in the interaction of these peptides with CALHM2 (Ext Data Fig. 4k).

## Discussion

CALHM2’s double barrel structure is striking and opened a door to identify a cellular role for this protein. When expressed in cells heterologously, previous reports described CALHM2’s electrophysiological properties, but these studies gave no deeper understanding of its cellular function. We have now discovered that CALHM2 resides on the matrix side of the inner mitochondrial membrane and is necessary for the import of a mitochondrial enzyme to the matrix. Although unexpected, this role is nevertheless consistent with its established structure. CALHM2 exemplifies a system for protein import into mitochondria that is divergent from the canonical TIM/TOM pathway.

The electrophysiological properties of CALHM2 may be predictive of its function in mitochondrial protein import. Our reconstituted proteoliposome and lipid bilayer single channel studies confirm that CALHM2 is inhibited by Ca^2+^. It is likely that CALHM2 is mostly in the closed state *in vivo*, since a frequently open pore in the inner mitochondrial membrane would abolish the proton gradient generated by the electron transport chain. The ECHA presequence is negatively charged, linear, and inhibits channel conductance (suggesting that it enters the pore), and the protein may reside in the pore during cell life, preventing a large leak from forming in the mitochondrial inner membrane. Importantly, our results also demonstrate that the ECHA peptide does in fact transit through the CALHM2 channel based on mass spectrometry on the *trans* side of the planar lipid bilayers. Once through, enzymes must chaperone the protein into its three-dimensional structure. Consistent with this idea, our co-IP mass spectrometry data identify two chaperones (HSP90 and HSP70), which further supports the function of CALHM2 as a protein import channel.

The mTFP is composed of two subunits, ECHA and ECHB. Interestingly, we find that ECHA and ECHB peptides interact differently with the CALHM2 channel. This finding is perhaps predicted by the decreased number and alternative positioning of charged amino acid residues and the non-linear structure of ECHB. The differential structure of the ECHB peptide may make it more difficult for this protein to inhibit the channel conductance. Indeed, in the presence of the ECHB peptide, we observed opening of the channel upon application of an increased voltage difference across the membrane, clearly differentiating between the efficiency of ECHA and ECHB in their effects on the channel. One intriguing possibility is that ECHB may help open the channel for ECHA transport. Future experiments will further clarify the role of ECHB in protein import to the matrix.

Mutations in either ECHA or ECHB result in metabolic diseases of mTFP deficiency^21,27–29^, and defects of ECHA result in long-chain 3-hydroxyacyl-CoA dehydrogenase deficiency (LCHAD)^30,31^. The activities of ECHA and ECHB are critical for survival and the disruption of their functions can lead to sudden death and severe cardiomyopathy^21,32^. However, a detailed analysis of the metabolic state of patients with disrupted mTFP function has been lacking.

We comprehensively analyzed the metabolic defects in CALHM2 depleted cells. We find that CALHM2 depletion results in mTFP deficiency, with phenotypes that highlight critical functions of ECHA, β-oxidation and cardiolipin-dependent mitochondrial inner membrane structure maintenance^21^. We find that loss of ECHA in the matrix alters cardiolipin amount, and cardiolipin is an essential component of mitochondrial cristae leading to abnormal cristae morphologies in CALHM2 depleted cells. Regarding energy metabolism, CALHM2 depleted cells have a dramatic reduction in β-oxidation, manifested as lipid accumulation in the cytosol, as expected from disruption of ECHA location. Unexpectedly, however, glucose-dependent TCA cycle activation is also disrupted with the loss of multiple TCA cycle enzymes. The resulting metabolic deficiencies include reduction in glycolytic capacity, reduction in TCA cycle and ETC components and activity. These metabolic changes result in reduced respiration and impaired cell mitosis. Interestingly, although cardiolipin is reduced with alterations in mitochondrial cristae, the impact on ETC protein levels which resides in these cristae seems less dramatic than on the TCA cycle enzymes which reside in the matrix. An exciting avenue for future study is the possible role of CALHM2 in the import of matrix enzymes such as those comprising the TCA cycle.

Finally, we originally identified CALHM2 in a patient with congenital heart disease and heterotaxy, suggesting it could have a role in embryonic patterning^33^. To this end, we previously showed that mitochondrial metabolism plays a significant role in establishing the vertebrate body plan^34^. This suggests that mitochondrial metabolism may have evolved to exploit bioenergetics to support multicellularity. Many of the foundational studies describing mitochondrial protein import have exploited the advantages of the eukaryotic system *S. cerevisiae*^9,35–40^. However, fatty acid oxidation in metazoans evolved differently. Yeast perform fatty acid oxidation in peroxisomes, whereas metazoans perform fatty acid oxidation in the mitochondrial matrix where it is tightly coupled to respiration. Therefore, unlike in yeast, proteins necessary for fatty acid oxidation in multicellular organisms must be imported across the two mitochondrial bilayers to enter the matrix. We propose CALHM2 as this evolutionary innovation in protein import.

## Materials and Methods

**Table:** 

REAGENT or RESOURCE	SOURCE	IDENTIFIER
**Antibodies**		
Rabbit polyclonal anti-CALHM2	Abcam	Cat#ab121466; RRID:AB_11139736
Mouse monoclonal anti-Tom20	Santa Cruz	Cat#sc17764 RRID:AB_628381
Mouse monoclonal anti-SOD2 (clone 2A1)	Abcam	Cat#ab16956 RRID: AB_302569
MCU	Cell Signalling	Cat#14997 RRID:AB_2721812
Mouse monoclonal anti-PDH	InVitrogen	Cat#459400 RRID: AB_ 1502002
Rabbit monoclonal anti-HADHA	Abcam	Cat#ab110302 RRID: AB_10862577
Mouse monoclonal anti-HAHDB	Abcam	Cat#ab110301 RRID: AB_10865743
VDAC	Cell Signalling	Cat#4866 RRID:AB_2272627
Mouse monoclonal anti-GAPDH	Ambion	Cat#AM4300 RRID: AB_437392
Mouse monoclonal anti-SERCA	Affinity BioReagents	Cat#MA3-919 RRID: AB_325502
Mouse monoclonal anti-Mitofilin	Abcam	Cat#ab110329 RRID: AB_10565198
Mouse monoclonal anti-ACSL4	Santa Cruz	Cat# SAB2100163, RRID:AB_10604940
Mouse polyclonal anti-PDH	ThermoFisher	Cat# 45-6799 RRID: AB_2533826
Mouse monoclonal anti-CS	ThermoFisher	Cat#MA5-17264 RRID: AB_2538732
Mouse monoclonal anti-SDHA	ThermoFisher	Cat# 459200 RRID: AB_10838019
Rabbit polyclonal anti-MDH2	ThermoFisher	Cat# PA5-21700 RRID:AB_11156233
**Chemicals, Peptides, and Recombinant Proteins**		
Protein A/G magnetic beads	Thermo Fisher	Cat#78609
Mitotracker^™^ Deep Red FM	Thermo Fisher	Cat#M22426
FCCP	Santa Cruz	Cat#370-86-5
Oligomycin	Sigma	Cat#75351
Rotenone	Sigma	Cat# R-8875
Antimycin A	Sigma	Cat# A8674
U-13C6 D-glucose	Cambridge Isotope Laboratories	Cat# INC CLM-1396
ECHA peptide MVACRAIGILSRFSAFRILRSRGYICRNFTGSSALL	LifeTein	
ECHB peptide MTILTYPFKNLPTASKWALRFSIRPLSCSSQLR	LifeTein	
COXIV peptide MLSLRQSIRFFKY	LifeTein	
**Critical Commercial Assays**		
ATP kit	Sigma	MAK135
Cardiolipin Assay Kit	Biovision	Cat#K944
Mitochondrial Extraction Kit	Qiagen	37612
Lipofectamine3000	Invitrogen	Cat#13778-075
FCCP	Santa Cruz	Cat#CAS
Bodipy	Thermo Fisher	Cat#D3922
		
**Deposited Data**		
PRIDE	Dataset Identifier: PXD010387	http://www.proteomeexchange.org/
		
**Experimental Models: Cell Lines**		
hTERT RPE-1	ATCC	CRL-4000
HEK-293 cells	ATCC	CRL-1573
**Oligonucleotides**		
*CALHM2* Human CRISPR sgRNA KD1 5’ GAGATGAACAGACCAGGTGAC	Sigma	This Paper
*CALHM2* Human CRISPR sgRNA KD2 5’ GGGCCGGCGAGCAGGGGCAG	Sigma	This Paper
**Recombinant DNA**		
Human CALHM2	Human ORFeomeV7.1	IMAGE:3509518
CALHM2 Myc-DDK-tagged	Origene	Cat#RC200512
**Software and Algorithms**		
Volocity 6.3 visualization and colocalization analysis	Perkin Elmer	Volocity 6.3 visualization and colocalization analysis

### Cell Culture

Human Tert-RPE1 (human telomerase-immortalized retinal pigmented epithelial; ATCC) cells were maintained in DMEM/Ham’s F12 supplemented with 10% fetal bovine serum (FBS), 1mM sodium pyruvate (Gibco), 1X non-essential amino-acids (Gibco) and 1X PEN/STREP antibiotics at 37°C and 5% CO_2_. Some cells were transfected with Lipofectamine 2000 (Life Technologies) by manufacturer’s instructions. Cells for mitochondrial extraction were grown to confluence in 100 mm dishes.

### Immunofluorescence

RPE cells were washed with 1XPBS and fixed in 0.20% Glutaraldehyde/4% PFA/0.1% TritonX for 20 sec. and then 10min with 4% PFA/ 0.1% TritonX. After fixation, cells were washed with PBS/0.5%Tween20 (PBST) blocked with 5% bovine serum albumin in PBST for 30min. Primary antibodies were diluted 1/300 in blocking buffer and cells were incubated with them for 1 hr at RT. After three washes with PBST for 5min each, secondary antibodies conjugated to Alexa 488, 568, 647 or Texas red (Thermo Fisher Scientific) were diluted 1/1000 in blocking buffer and incubated for 30 min. Alexa647 phalloidin (Molecular Probes 1:50) was used to stain cell actin. Mammalian cells were mounted on coverslips using Prolong Gold antifade reagent (Life Technologies). Hoescht (333423, Life Technologies) was used to stain DNA.

### Western Blots

Protein concentrations were quantified using DC Protein Assay (Biorad). Western blotting was performed using standard protocols and 40 μg was loaded onto a polyacrylamide gel. GAPDH was used as a loading control for whole cell extracts. For mitochondrial purifications, VDAC was used as a loading control. For protein detection, we used anti-mouse or anti-rabbit HRP conjugated secondary antibodies (Jackson Immuno Research Laboratories) and Western Lightning Plus ECL (Perkin Elmer).

### Generation of CALHM2 knockout RPE cells by CRISPR/Cas9

Two guide RNAs (gRNAs) specifically targeting *CALHM2* exon 3 were selected from a list designed using the E-CRISP website (http://www.e-crisp.org/E-CRISP/designcrispr.html) from the German Cancer Research Center. The selected sequences were: #16: 5’ GAGATGAACAGACCAGGTGAC; and #34: 5’GGGCCGGCGAGCAGGGGCAG3’. Oligos for these sequences were annealed and ligated to the LentiCRISPRv2 plasmid that was cut with BsmBI as described ^41^. Lentivirus was produced with these plasmids as recommended on the Addgene website, and used to infect RPE cells. Forty-eight hours after infection, RPE cells were selected with 10 μg/mL of Puromycin. Puromycin resistant cells were replated and used to isolate single clones by serial dilution in 96-well plates.

### Structured Illumination Microscopy (SIM)

Images were acquired using a U-PLANAPO 60X/1.42 PSF, oil immersion objective lens (Olympus, Center Valley, PA) and CoolSNAP HQ^2^ CCD cameras with a pixel size of 0.080μm (Photometrics, Tucson, AZ) on the OMX version 3 system (Applied Precision) equipped with 488-, 561-, and 642-nm solid-state lasers (Coherent and MPB communications). Samples were illuminated by a coherent scrambled laser light source that had passed through a diffraction grating to generate the structured illumination by interference of light orders in the image plane to create a 3D sinusoidal pattern, with lateral stripes approximately 0.270 nm apart. The pattern was shifted laterally through five phases and through three angular rotations of 60° for each Z-section, separated by 0.125 nm. Exposure times were typically between 75 and 150 ms, and the power of each laser was adjusted to achieve optimal intensities of between 1,000 and 3,000 counts in a raw image of 12-bit dynamic range, at the lowest possible laser power to minimize photo bleaching. Raw images were processed and reconstructed using Softworx software (GE healthcare) to reveal structures with 100–125 nm resolution^42^. The channels were then aligned in x, y, and rotationally using predetermined shifts as measured using a target lens and the Softworx alignment tool.

### Expansion microscopy (pan-ExM)

Tissue expansion was performed as previously described (Panluminate, Inc)^43^. Briefly, kidney cells were incubated in a solution of acrylamide and fomaldehyde. After fixation, the cells were embedded in the expansion gel solution and then placed in MilliQ water for expansion. Gels were then re-embedded and the process was repeated until the desired expansion was achieved at which point antibody labeling and pan-staining were performed as described^43^.

### Mitochondrial and mitochondria-associated ER membranes (MAMs)

MAMs and mitochondria were isolated from kidneys of adult C57BL/6 mice as previously described^44^. For outer mitochondrial membrane solubilization, the isolated mitochondria were resuspended in PBS supplemented with 250 mM mannitol with or without digitonin (2 and 4 mg/ml). Samples were vortexed in a multi-vortex at room temperature for 15 minutes and centrifuged at 10,000 g for 10 minutes. The pellet of mitochondria was resuspended in loading buffer for western blot analysis. Digitonin was prepared as a 40 mg/ml stock solution in water.

### Isolation of mitochondria from RPE cells.

Mitochondria were isolated from RPE wild-type and CALHM2 KD cells. In brief, cells were transferred to ice-cold isolation buffer (250 mM sucrose, 20 mM Hepes (pH 7.2), 1 mM EDTA, and 0.5% BSA), supplemented with 1x Halt protease inhibitor. Cells were minced, homogenized with a Dounce homogenizer, and centrifuged at 1000 × g to pellet nuclei, cell debris and unbroken cells. The supernatant was centrifuged at high-speed (6000 × g for 15 min at 4 °C); the pellet containing mitochondria was washed in isolation buffer and pelleted by centrifugation at 6000 × g. The isolated mitochondria were kept on ice and used within 4 h. Protein concentration was determined by the BCA method using bovine serum albumin (BSA) as a standard.

### Immunoprecipitation of human CALHM2 protein for Mass Spec

Human CALHM2 Myc-DDK-tagged ORF clone (Origene Technologies, RC200512) was expressed in HEK 293T cells and overexpression was verified by Western blot analysis using a mouse anti-Myc antibody (Cell Signaling Technology). Mitochondria overexpressing CALHM2-Myc-DDK protein were isolated from HEK cells and solubilized with 1 mM N-dodecyl maltoside for 30 min on ice. The solubilized mitochondria were centrifuged for 5 min at 16,000 × g to remove any remaining membrane fragments. To IP Myc-DDK-tagged CALHM2, EZview Red ANTI-FLAG M2 Affinity Gel (Sigma) was added at a dilution of 1:100 and incubated with gentle agitation for at least 2 hr at room temperature or overnight in the cold room. The beads were then washed twice before being treated with Myc-peptide to elute Myc-DDK-tagged CALHM2. The eluate fraction was analyzed with LC-MS mass spectrophotometry to identify the proteins interacting with CALHM2. Another Myc-DDK-tagged protein (ATP synthase c-subunit) was used as a negative control to rule out the possibility of non-specific binding of identified proteins to Myc-DDK tag. The mass spectrometry proteomics data have been deposited to the ProteomeXchange Consortium via the PRIDE partner repository with the dataset identifier PXD010387 ^45^.

### CoIP of CALHM2 and mTFP subunits

Cells were collected in ice-cold PBS and then lysed in NP-40 buffer (150 mM NaCl, 1.0% NP40, 50 mM Tris, pH 8.0). In order to reduce non-specific binding, Protein A beads were then added to the sample and incubated overnight at 4°C. Protein A beads were then magnetized to isolate the supernatant. The supernatant was incubated with primary antibody overnight at 4°C. New Protein A beads were added and then magnetized. Beads were washed 3 times in PBS. Then 2x SDS loading dye was added and heated to 95°C for five mins. Samples were then analyzed by western blot.

### Electron Microscopy

Cells were fixed in 2.5% glutaraldehyde in 0.1M sodium cacodylate buffer, pH 7.4 for 1 hr. Buffer rinsed cells were scraped in 1% gelatin and spun down in 2% agar. Chilled blocks were trimmed and postfixed in 1% osmium tetroxide for 1 hr. The samples were rinsed three times in sodium cacodylate rinse buffer and postfixed in 1% osmium tetroxide for 1 hr. Samples were then rinsed and en bloc stained in aqueous 2% uranyl acetate for 1 hr followed by rinsing, dehydrating in an ethanol series, infiltrated with Embed 812 (Electron Microscopy Sciences) resin, and then baked overnight at 60°C. Hardened blocks were cut using a Leica UltraCut UC7. Sections (60 nm) were collected in formvar/carbon-coated nickel grids and contrast stained with 2% uranyl acetate and lead citrate. They were viewed using a FEI Tencai Biotwin TEM at 80Kv. Images were taken on a Morada CCD using iTEM (Olympus) software.

### Neutral lipid staining and measurement

Neutral lipid staining was performed using BODIPY (Invitrogen). Cell were washed 3 times in PBS, cells were then incubated in a solution of BODIPY in DMSO and diluted 1:200 in PBS. Cells were then fixed in 4% PFA/PBS and were observed by confocal microscopy to monitor fluorescence levels at Ex/Em = 489/503 nm. The fluorescence intensities of the stained cells were quantified using ImageJ.

### Measuring Cardiolipin Content

Mitochondria were prepared as previously described. Cardiolipin content of mitochondria was quantified using the Cardiolipin Assay Kit (Biovision #K944). Briefly, 20 mg of mitochondria was added to a 96-well white plate and a total volume of 50 μl was added to the well with Cardiolipin buffer and probe. A background control was prepared using 100ml cardiolipin buffer. The samples were incubated for 10 minutes at room temperature. Cardiolipin content was measured at Ex/Em 340/480nm using a Victor 3 plate reader (Perkin Elmer) and correlated to the standard curve with known amounts of cardiolipin after subtraction of background.

### Determination of mitochondrial ATP content.

Luminescent ATP detection kit (Sigma-Aldrich) was used for determination of ATP level in wild-type and CALHM2 knock-down mitochondria. The working reagent lyses mitochondria to release ATP, which then interacts with added firefly’s luciferase and luciferin to produce light. The light intensity was a direct measure of the mitochondrial ATP content. ATP-induced luminescence was measured with a Victor 3 plate reader (Perkin Elmer).

### Cell Growth Assay

RPE wild-type and CALHM2 knock-down cells were plated at 35,000 cells/mL concentration into 6-wells plate and incubate over-night at 37°C. The next day the cells were washed with fresh complete media and placed into an Evos FL2 auto equipped with an onstage incubator set for 37°C and 5%CO_2_ (Thermo Fisher). Nine different fields for wild-type and CALHM2 KD wells cells were imaged every 20min with brightfield phase contrast using a 20X Olympus objective for a total of 36hr. The time-lapse data for each cell line was imported into Imaris software 10.1 (Oxford instruments) for cell segmentation to identify and quantify the number of mitotic cells per hr during the 36hr period.

### Agilent XF96 Pro Respirometry

50,000 RPE cells per well were plated in an Agilent XF96 cell culture plate 24 hours prior to the respirometry measurements on an Agilent Technologies XF96 Pro Analyzer. One hour before the study, RPE cells were washed and incubated at 37°C in DMEM (Sigma D5030) media supplemented with 2.0 mM Glutamine, 10 mM HEPES and 0.2% fatty acid free BSA, pH 7.4. Oxygen consumption rates (OCR) and extracellular acidification rates (ECAR) were measured in accordance with manufacturer’s instructions unless otherwise indicated (Agilent Technologies formerly Seahorse Bioscience). Basal oxygen consumption measurements (8 cycles) were made followed by an acute injection of either 5mM Glucose or 5mM Pyruvate compared to 1x assay media control injection (12 cycles). After the acute respiration recordings, mitochondrial oxidative function and acidification were first tested with the addition of oligomycin A (5 mM) [Sigma], an ATP synthase inhibitor. To induce maximal respiration, the proton uncoupler Trifluoromethoxy carbonylcyanide phenylhydrazone [Sigma] (FCCP, 20 mM) was injected. Finally, a mixture of antimycin A [Sigma] (10 mM) and rotenone [Sigma] (5 mM), an inhibitor of complex III and I respectively, was injected to shut down electron transport and assess non-mitochondrial oxygen consumption. Each respirometry cell well was normalized using Hoechst 33342 stain [Life Technologies] using the XF Pro and Cyt5 imaging integrated system (Agilent Technologies formerly Biotek).

### MIMOSA - LC-MS/MS Analysis

Metabolite concentrations and ^13^C-enrichments were determined by mass spectrometry using a SCIEX 5500 QTRAP equipped with a SelexION for differential mobility separation (DMS). Samples were injected onto a Hypercarb column (3 μm particle size, 3×150 mm, Thermo Fisher Scientific) at a flow rate of 1 mL/min. Metabolites were eluted with a combination of aqueous (A: 15mM ammonium formate and 10 μM EDTA) and organic mobile phase (B: 60% acetonitrile, 35% isopropanol and 15mM ammonium formate) according to the following gradient: t=0min, B=0%; t=0.5min, B=0%, t=1min, B=40%; t=1.5min, B=40%; t=2min, B=0%; t=6min, B=0%. Metabolite detection was based on multiple reaction monitoring (MRM) in negative mode using the following source parameters: CUR: 30, CAD: high, IS: −1500, TEM: 625, GS1: 50 and GS2: 55. DMS parameters were DT: low, MD: 2-propanol, MDC: low, DMO: 3 and DR: off, while Separation Voltage (SV) and Compensation Voltage (CoV) were optimized individually for each metabolite in order to maximize signal intensity and isobar resolution. The individual MRM transition pairs (Q_1_/Q_3_) are listed in Table S1. Retention times were confirmed with known standards and peaks integrated using El-Maven. The atomic percent excess (APE) was calculated using Polly interface and corrected for background noise and for natural abundance (Elucidata Corporation). Endogenous taurine, an intracellular osmolyte, was used as internal control for cell density as previously described ^46^.

### Steady-State Flux Ratio

$V_{PDH}/V_{CS}$ was calculated according to equation 1. Acetyl-CoA and oxaloacetate enrichments were calculated based on the deconvolution of citrate mass isotopomers applied to MRM Q_1_/Q_3_=191/111^24^. The sources of [M+3] malate were distinguished based on the enrichment ratio between succinate and malate and validated by comparison with oxaloacetate enrichments ^24^.


(Eq. 1) $$\frac{V_{PDH}}{V_{CS}}=\frac{[M+2]\mathrm{acetyl}-\mathrm{CoA}}{[M+3]PEP}$$

### Purification of human CALHM2 protein for electophysiology

Myc-DDK-tagged CALHM2 plasmid was overexpressed in HEK 293T cells for 48 hours followed by the mitochondria isolation. n-dodecyl-ß-D-maltoside (DDM) -solubilized mitochondrial lysate was incubated with the EZview Red ANTI-FLAG M2 Affinity Gel beads for 2h then the beads were washed extensively to remove non-specifically bound proteins. Myc-peptide was used to elute Myc-DDK-tagged CALHM2 protein.

### Patch clamp and planar lipid bilayer electrophysiology

The patch-clamp recordings of CALHM2-reconstituted proteoliposomes were performed by forming a giga-ohm seal in intracellular solution (10 mM Hepes, pH 7.3, 120 mM KCl, 8 mM NaCl, 0.5 mM EGTA,) using an Axopatch 200B amplifier (Axon Instruments) at room temperature (22–25 °C). Recording electrodes were pulled from borosilicate glass capillaries (WPI) with a final resistance in the range of ~50 MΩ. Signals were filtered at 5 kHz using the amplifier circuitry.

Proteoliposomes were prepared according to published protocols^47,48^. Briefly, 50 mg of phosphatidylcholine (Sigma) was dissolved in 1 mL of chloroform. A thin lipid film was formed on a glass surface by evaporating the chloroform. Liposomes were formed by the reconstitution of the lipid in rehydration buffer containing 250 mM KCl, 5 mM HEPES, and 0.1 mM EDTA. Then, 20 μg of recombinant CALHM2 protein was added to 100 μL of the liposome mixture (∼2 mg of lipids, final), and the samples were vortexed twice. Ca^2+^ (7.5 mM, final concentration) and ECHA or ECHB peptides (5 μg, final concentration) were added into the bath during the recordings without perfusion.

Planar lipid bilayer recordings were performed in intracellular solution by using α-L-phosphatidylcholine (Sigma) for forming the bilayer membrane. ePatch amplifier (Elements) was used for lipid bilayer recordings. ECHA or ECHB peptides (5 μg, final concentration) were added on the *cis* side of the cuvette during the recordings without perfusion. Purified CALHM2 was added on the *cis* side and a constant voltage was applied to achieve protein insertion into the bilayer.

For proteoliposomes and planar lipid bilayer recording, to access the channel activity at negative and positive voltages, a voltage ramp was performed where the voltage was changed from −100 to +100 mV within 60 seconds. For data analysis, Clampfit software (Molecular Devices) was used. The measured current was adjusted for the holding voltage assuming a linear Current-Voltage relationship. The conductance (G) is expressed in pS, following equation *G = I/V*, where *I* is the peak membrane current in pA and *V* is the membrane holding voltage in mV. Group data were quantified in terms of peak conductance and probability of channel opening, where NPo is the number of open channels (“level” in pCLAMP) times the probability of channel opening at each level. All population data were expressed as mean ± SEM.

### MALDI TOF

MALDI TOF MS Flex instrument (Bruker Tims Tof) was used to assess the molecular weight of peptides. The matrix (CHCA - alpha-cyano-4-hydroxycinnamic Acid, 5mg) was mixed with the 1 ml solvent (490ul H_2_O, 500 μl Acetonitrile, 10 μl of 10% TFA in H_2_O).

The matrix was then mixed with the solution from the *trans* side of bilayer cuvette (4:1), and the sample (2 μl) was deposited on a 384 polished steel sample plate. A MALDI 20000 method enabling detection of peptides with a size of up to 20000 Da was used.

### Structural Analysis

The three-dimensional models of ECHA and ECHB N-terminal signal peptides were generated by AlphaFold^49^. The electrostatic potential maps of peptides were generated using APBS-PDB2PQR software.

### Statistical Analysis

All experiments were conducted with a minimum of three replicates, and the numbers reported in the graphs reflect data from multiple experimental runs. Data were tested for normality using the Shapiro-Wilk test where sample sizes permitted, which informed the selection of appropriate parametric or nonparametric statistical methods. For normally distributed data, results are expressed as means ± SEM. Statistical comparisons between two experimental groups were performed using a two-tailed t-test. For comparisons involving multiple groups, one-way analysis of variance (ANOVA) was utilized, with Fisher’s post hoc test applied to account for multiple comparisons. The homogeneity of variances across groups was assessed using Levene’s test. When Levene’s test indicated equal variances, a standard t-test was employed. In cases where Levene’s test revealed unequal variances, Welch’s t-test was used instead. For data that did not follow a normal distribution, the Mann-Whitney U test was applied. Post-hoc comparisons were adjusted for unequal variances using Dunn’s test with Bonferroni corrections. Statistical significance was defined as p < 0.05 in all figures. For co-localization experiments using immunofluorescence, Pearson correlation coefficients of the different channels were calculated using Volocity 6.3 software (Perkin Elmer).
